# The dual effect of a ferredoxin-hydrogenase fusion protein in vivo: successful divergence of the photosynthetic electron flux towards hydrogen production and elevated oxygen tolerance

**DOI:** 10.1186/s13068-016-0601-3

**Published:** 2016-08-30

**Authors:** Haviva Eilenberg, Iddo Weiner, Oren Ben-Zvi, Carmel Pundak, Abigail Marmari, Oded Liran, Matt S. Wecker, Yuval Milrad, Iftach Yacoby

**Affiliations:** 1 Department of Molecular Biology and Ecology of Plants, The George S. Wise Faculty of Life Science, Tel Aviv University, Ramat Aviv, 69978 Tel Aviv, Israel; 2 GeneBiologics, LLC, Boulder, CO USA; 3 National Renewable Energy Laboratory, Golden, CO USA

**Keywords:** H_2_ production, Ferredoxin, Hydrogenase, Oxygen sensitivity, Fusion enzyme, *Chlamydomonas reinhardtii*

## Abstract

**Background:**

Hydrogen photo-production in green algae, catalyzed by the enzyme [FeFe]-hydrogenase (HydA), is considered a promising source of renewable clean energy. Yet, a significant increase in hydrogen production efficiency is necessary for industrial scale-up. We have previously shown that a major challenge to be resolved is the inferior competitiveness of HydA with NADPH production, catalyzed by ferredoxin-NADP^+^-reductase (FNR). In this work, we explored the in vivo hydrogen production efficiency of Fd-HydA, where the electron donor ferredoxin (Fd) is fused to HydA and expressed in the model organism *Chlamydomonas reinhardtii.*

**Results:**

We show that once the Fd-HydA fusion gene is expressed in micro-algal cells of *C. reinhardtii*, the fusion enzyme is able to intercept photosynthetic electrons and use them for efficient hydrogen production, thus supporting the previous observations made in vitro. We found that Fd-HydA has a ~4.5-fold greater photosynthetic hydrogen production rate standardized for hydrogenase amount (PHPRH) than that of the native HydA in vivo. Furthermore, we provide evidence suggesting that the fusion protein is more resistant to oxygen than the native HydA.

**Conclusions:**

The in vivo photosynthetic activity of the Fd-HydA enzyme surpasses that of the native HydA and shows higher oxygen tolerance. Therefore, our results provide a solid platform for further engineering efforts towards efficient hydrogen production in microalgae through the expression of synthetic enzymes.

**Electronic supplementary material:**

The online version of this article (doi:10.1186/s13068-016-0601-3) contains supplementary material, which is available to authorized users.

## Background

As hydrogen gas is a clean energy carrier, it is a sound candidate for the replacement of fossil fuels. Though hydrogen is the most abundant element in the universe, gaseous hydrogen is scarce on earth because it is light enough to escape earth’s gravity and is therefore naturally lost to outer space [1].

Hydrogen gas production occurs in certain microorganisms that catalyze its production through two enzyme families: nitrogenases that fix atmospheric nitrogen into ammonia while emitting gaseous hydrogen as a byproduct, and hydrogenases that either produce or oxidize hydrogen directly. Algal [FeFe]-hydrogenase (hereafter HydA) is of great industrial interest due to its extremely high turnover rate. Typical numbers measured in chemical reactions with an artificial reduced electron donor such as methyl viologen (MV) reach remarkable rates of up to 10^4^ hydrogen molecules per second [2]. However, HydA is extremely sensitive to oxygen, which is a direct product of photosynthesis. The enzyme’s typical half-life under aerobic conditions ranges from a few seconds for algal HydA to several minutes for the *Clostridia* [FeFe]-hydrogenase, which contains additional iron sulfur clusters [3, 4]. Therefore, oxygen sensitivity of HydA constitutes a primary technological barrier to overcome in order to make commercial production of hydrogen feasible.

Photo-biological hydrogen production is carried out by microalgae that naturally express HydA as a defense mechanism against excess electrons. A heightened electron flux that requires this sort of defense is formed in anaerobiosis and light (e.g., under sulfur deprivation) [5]. The reducing power that feeds HydA activity originates mainly from water splitting at the photosystem II (PSII) complex [6]. Notably, the electron flux throughout the electron transport chain, embedded in the thylakoid membrane, has several ‘stations’ en route to HydA; the last and most important of which is the electron transfer from the photosystem I (PSI) complex to ferredoxin (Fd). The electron flux from PSI to the majority of downstream electron acceptors is principally mediated by Fd, which serves as an electron carrier. Furthermore, besides reducing HydA, Fd additionally reduces other electron acceptors [7, 8], primarily ferredoxin-NADP^+^-oxidoreductase (FNR) [7, 9]. This enzyme catalyzes NADPH production, which supplies reducing equivalents for growth-essential carbon fixation in the Calvin–Benson pathway. Therefore, evolutionary pressure supports mechanisms that ensure superior electron transport towards FNR and NADPH production at the expense of other competing processes. Indeed, we have shown that more than 85 % of the electron flux is naturally directed to NADPH production [9].

Previous evidence [10–12] shows a physical localization of FNR at PSI. Initial evidence for FNR direct docking at the PSI subunit psaE was provided more than 20 years ago by Andersen et al. [10]. This discovery was recently strengthened by the works of Iwai et al. [11] and Takahashi et al. [13], who showed that FNR participates in a mega CEF complex that is formed around PSI. In our previous work [9], we fused Fd to hydrogenase to form the Fd-HydA. Importantly, we showed that the Fd-HydA alone has no photosynthetic activity, i.e., Fd-HydA’s ferredoxin is not involved in electron transfer from PSI to the fused hydrogenase. Only an external addition of free Fd results in Fd-HydA photosynthetic activity. However, when tested in a competition assay against FNR, Fd-HydA significantly outperforms the native HydA. The conclusion of this paper is that the fused Fd moiety functions as an anchor bringing HydA closer to PSI, rather than an electron mediator. In other words, by simply bringing hydrogenase closer to the electron source, its competition abilities increase greatly. Thus, spatial localization has a major role in determining the electron partitioning and transfer rate between electron acceptors downstream of PSI.

Several engineering efforts were made to bypass this FNR superiority. For example, Winkler et al. recently observed that a redesigned Fd could support a fivefold hydrogen production rate at the expense of FNR activity [7]. In their work, electron divergence towards HydA was achieved by limiting the binding capacity of the redesigned Fd to FNR while leaving the binding efficiency to HydA intact. However, the electron donor was a small chemical photosynthesizer molecule rather than a purified PSI complex; hence, the effect of FNR binding to PSI was not studied. In another route, in vivo silencing of FNR resulted in a 2.5-fold increase in the measured hydrogen production rate under sulfur deprivation [14]. In our previous work [9], we showed that by substituting HydA for Fd-HydA fusion protein in in vitro reconstituted reactions, we diverted 60 % of the electron flux towards hydrogen production, while limiting NADPH production. However, we did not investigate whether Fd-HydA could perform effectively in vivo.

In this work, we explored the ability of Fd-HydA to intercept the electron flux in vivo in the model organism *Chlamydomonas reinhardtii.* Specifically we studied the following questions in vivo:

1. Is the intrinsic PHPRH of Fd-HydA greater than that of the native HydA?
2. Based on the fused iron cluster of Fd, is the oxygen sensitivity of Fd-HydA altered?

## Results

### Isolation of transgenic H_2_ producing algal strains

To study the in vivo H_2_ production rate of the Fd-HydA fusion, we first obtained a *C. reinhardtii* strain with negligible hydrogen production background, i.e., *hydA1*-*1 hydA2*-*1* double mutant (*hyd*A_1,2_) that was derived from the parental strain D66 [15]. We then created expression platforms harboring a *C. reinhardtii* codon-optimized Fd-linker ([Gly_4_-Ser]_3_)-HydA sequence, placed under the control of (a) the hybrid Hsp70A-RbcS2 promoter [16] in the commercial vector pChlamy_1 (GeneArt); (b) the psaD promoter [17] in the pSL18 vector or (c) the endogenous *C. reinhardtii* hydA1 promoter (a kind gift from Prof. Maria Ghirardi of NREL) (all are schematically illustrated in Additional file 1, panels a–c). These constructs were then separately transformed into the nuclei of *hyd*A_1,2_ algal cells.

**Fig. 1:**
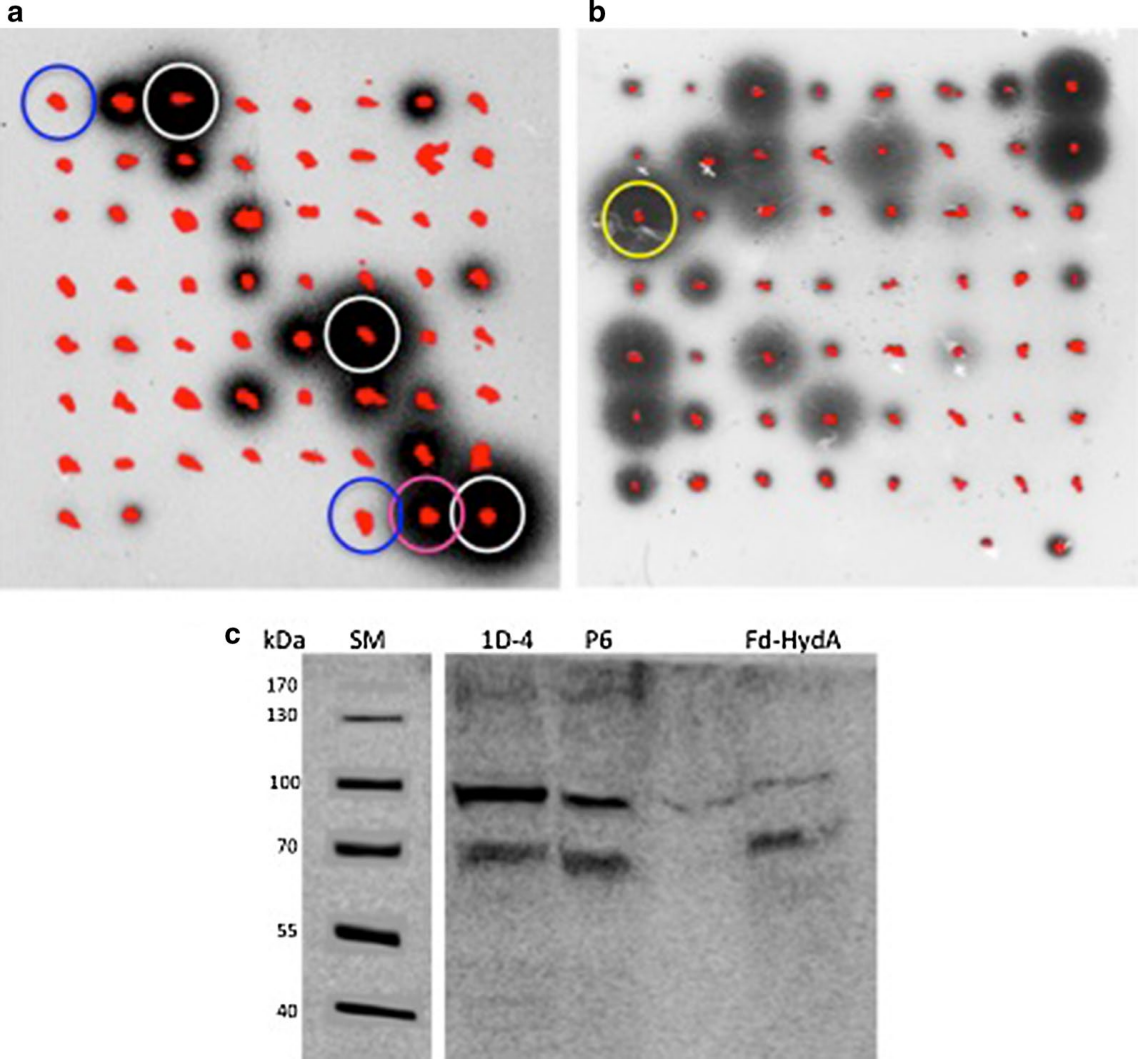
Screening for Fd-HydA expressing clones. *C. reinhardtii hydA*_*1,2*_ double mutant cells were transformed with Fd-HydA either under **a** Hsp70-RbcS2 or **b** psaD promoter. The transformants were screened for gene expression using the H_2_ sensitive *R. capsulatus* screen. Chlorophyll fluorescence of the algae is shown in *red* whereas *dark halation* represents GFP produced by *R. capsulatus* upon H_2_ presence. The wt strain CC-124 (*white circle*) and *hydA*_*1,2*_ double mutant (*blue circle*) clones were used as positive and negative controls, respectively. The highest Fd-HydA expressing clones (1D4 for Hsp70-RbcS2 and P6 for psaD) are *circled* with *purple* and *yellow*, respectively. **c** Immunoblot analysis for detection of Fd-HydA expression. Soluble proteins (75 µg of each) from 1D4 (*lane 1*) and P6 (*lane 2*) were loaded on 4-12 % Bis–Tris PAGE (Life Technologies) and probed with rabbit polyclonal HydA1/2 antibodies. *C. reinhardtii* Fd-HydA (10 ng) expressed heterologously and purified from *E. coli* (co-loaded with soluble protein from the *hydA*_*1,2*_ mutant) was used as positive control (*lane 4*)

The high-throughput *Rhodobacter capsulatus* screening system [18, 19] was used for recovering successful H_2_-producing clones. In this method, the H_2_ sensing system of *R. capsulatus* bacteria was engineered to express GFP when sensing molecular hydrogen in its immediate proximity. After screening 100–250 colonies for each promoter group, several clones expressing Fd-HydA under the Hsp70A-RbcS2 and psaD promoters were successfully isolated (Fig. 1a, b), whereas the hydA1 promoter yielded no positive clones (Additional file 2). The isolated clones were then tested by gas chromatography for photosynthetic H_2_ production, following 2 h of dark anaerobiosis, as described (see methods). From each promoter group, we selected the clone with the highest photosynthetic hydrogen production rate; 1D4 of the Hsp70A-RbcS2 group and P6 of the psaD group, for further studies.

The fusion protein expression in the transformants was then verified by immunoblotting using a commercial (AgriSera) polyclonal antibody against HydA1/2 (Fig. 1c). This antibody recognizes the Fd-HydA fusion protein (~70 kDa) as well as the wild type (wt) HydA (55 kDa). As a positive control we used Fd-HydA or HydA heterologously expressed and purified from *Escherichia coli* [20]. These purified proteins include a StreptagII purification tag along with a small linker, thus explaining their higher migration in the gel. To produce comparative migration conditions of the purified standards and mimicking masking of the Fd-HydA signal by the *Chlamydomonas* extracts, we co-loaded the heterologous protein standards with *Chlamydomonas* extracts isolated from the parental double mutant *hyd*A_1,2_ lacking HydA protein. It should be noted that the commercial polyclonal antibody against HydA1/2 recognizes in addition to the specific HydA/Fd-HydA also another unspecific, ~100 kDa band in all *Chlamydomonas* extracts, including *hyd*A_1,2_ which lacks HydA protein.

### In vivo H_2_ production rates

Hydrogen production of 1D4 (Hsp70A-RbcS2 promoter), P6 (psaD promoter), the parental wt strain D66 and the wt strain CC-124 was measured following 120 min of dark anaerobic incubation (dark anaerobiosis was found to be superior to anaerobic incubation in light for all promoters, Additional file 3). It should be noted that maximal photosynthetic hydrogen production rates for wt and the fusion transformants were observed after 2 h of dark incubation under anaerobic conditions and therefore we used that induction time (Additional file 4). In order to verify that during the dark incubation period starch degradation rates in the different clones are similar, we measured the starch content of all cell cultures before and after dark anaerobiosis. Indeed, the results show no significant difference in degradation rates across the different clones (Additional file 5).

For each clone, samples were taken in parallel for an in vivo photosynthetic hydrogen production assay (see “Methods” section), showing the actual in vivo hydrogen production under moderate illumination (300 μE m^−2^ s^−1^) (Table 1) and for the determination of the total cellular content of active hydrogenase (HydA or Fd-HydA) (Table 1). In this protein quantification technique, hydrogen production rate is measured by forming a reaction between cell lysates and the artificial electron donor MV. This strong reductant is added to the reaction in great excess to efficiently reduce the entire pool of active HydA or Fd-HydA. Thus dividing the rate of chemically measured H_2_ production by the known specific activity of the enzymes, 1000 U/mg for native HydA purified from the algae [21] or expressed and purified heterologously [20] and 3500 U/mg for the Fd-HydA [20], yields the quantity (µmol or ng) of total active enzymes per mg chlorophyll.

**Table 1:** Comparison of hydrogenase activity in wt (CC-124 and D66) and Fd-HydA transformants (1D4 and P6)

	D66 (HydA)	CC-124 (HydA)	1D4 (Fd-HydA)	P6 (Fd-HydA)
Photosynthetic H_2_ production [µmol(H_2_) mg(chl)^−1^ h^−1^]	8.34 ± 1.4	21.65 ± 1.5	0.91 ± 0.027	6.06 ± 0.538
Chemical H_2_ production [µmol(H_2_) mg(chl)^−1^ h^−1^]	37.68 ± 8	104.6 ± 14.4	4.5 ± 0.55	26.9 ± 0.298
Amount of enzyme [ng(enzyme) mg(chl)^−1^]	628	1743	21	128
Amount of enzyme [µmol(enzyme) mg(chl)^−1^]	1.31 × 10^−5^	3.63 × 10^−5^	0.35 × 10^−6^	2.14 × 10^−6^
Photosynthetic H_2_ production rate standardized for hydrogenase amount (PHPRH) [µmol(H_2_) µmol(enzyme)^−1^ min^−1^]	10,622	9931	42,909	47,236
Ratio of PHPRH (transformant/wt average)	~1	~1	4.2	4.6

All of the experiments were performed in biological triplicates

The validity of the MV-based protein quantification technique was confirmed by determining protein levels for D66 (expressing HydA) and P6 (expressing Fd-HydA) by immunoblots in which purified standards of HydA and Fd-HydA were used. Here, the cultures underwent the same 120-min dark anaerobic incubation as mentioned above. For each strain, a single cell culture following 120 min of dark anaerobic incubation was divided into 2 samples: one which was taken for MV-based protein quantification and another which was taken for protein quantification by immunoblotting. The results (Additional file 6) show high similarity between protein levels determined by the 2 different methods. Thus, the MV-based protein quantification technique is highly reliable and was used for determination of total HydA or Fd-HydA proteins in other clones.

Based on the absolute quantification of the in vivo contents of HydA or Fd-HydA, the in vivo PHPRH could now be calculated by dividing the in vivo measured photosynthetic H_2_ production rate by the number of enzyme µmoles. Table 1 shows that the two wt strains, CC-124 and D66, although varying in protein expression levels, have similar PHPRH of ~10,000 µmol(H_2_) µmol(HydA)^−1^ min^−1^. Similarly, the two Fd-HydA transformants, although they differ in their expression profiles (clone P6 produces 6 times more protein than 1D4), both show similar PHPRHs at ~45,000 µmol(H_2_) µmol(Fd-HydA)^−1^ min^−1^.

### O_2_ tolerance of Fd-HydA

**Fig. 2:**
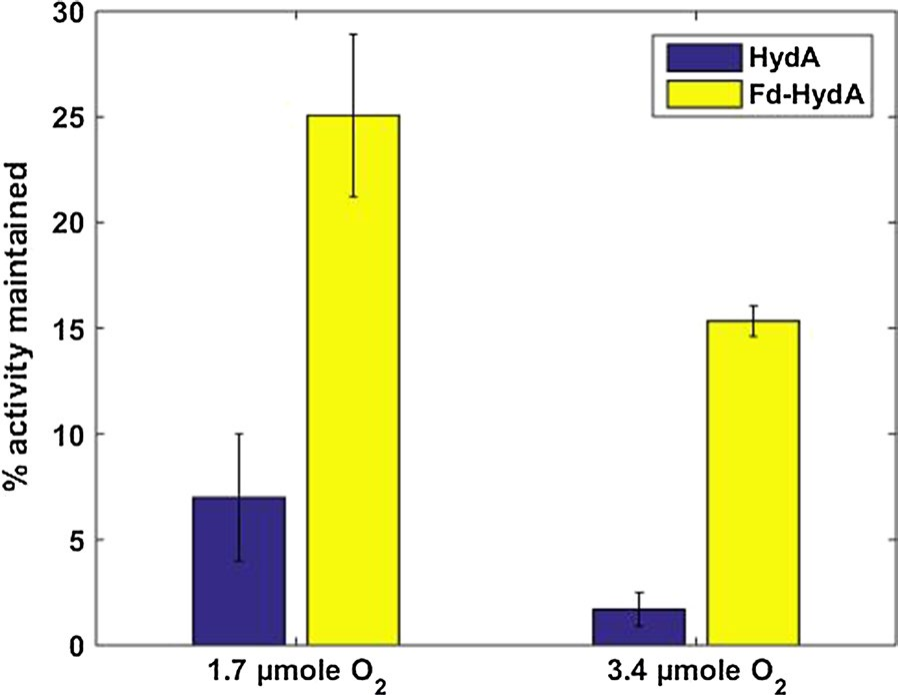
Elevated oxygen tolerance of the fusion enzyme in vitro. Survival percentage of 40 nM of purified HydA or Fd-HydA, upon 10 min of exposure to 2 different oxygen concentrations: 1.7 or 3.4 µmol. The hydrogenase activity after exposure to O_2_ was divided by the activity of the matching unexposed enzyme. Mean rates measured—unexposed Fd-hydA: 63.8 µmol(H_2_) mg(hydA)^−1^ min^−1^, Fd-HydA+ 1.7 µmol(O_2_): 16 µmol(H_2_) mg(hydA)^−1^ min^−1^, Fd-HydA+ 3.4 µmol(O_2_): 9.7 µmol(H_2_) mg(hydA)^−1^ min^−1^. Unexposed HydA: 66 µmol(H_2_) mg(hydA)^−1^ min^−1^, HydA+ 1.7 µmol(O_2_): 4.9 µmol(H_2_) mg(hydA)^−1^ min^−1^, HydA+ 3.4 µmol(O_2_): 1.2 µmol(H_2_) mg(hydA)^−1^ min^−1^

The *Clostridia* multi-[FeS]-cluster hydrogenase is significantly less vulnerable to atmospheric oxygen than the *Chlamydomonas* HydA [4]. In addition, it is known that Fd can reduce molecular oxygen to oxygen superoxide [22] that unlike molecular oxygen is charged and thus, cannot penetrate into the active site of HydA, the H cluster. This led us to question whether our Fd-HydA, harboring a [2FeS] cluster originating in the fused Fd, might prove less vulnerable to oxygen in vitro and in vivo when expressed in *C. reinhardtii*. To investigate this hypothesis we heterologously expressed and isolated *Chlamydomonas* HydA and Fd-HydA in *E. coli*, as described previously [20]. From each batch of purified proteins three samples (each of the 6 samples was properly diluted to receive the same final molar concentration of 40 nM [see “Methods” section]), were placed in septum-sealed 14 mL Wheaton vials and purged with Argon for 10 min to remove all O_2_. In each group one vial was injected with 200 μL of air (1.7 μmol O_2_), another with 400 μL (3.4 μmol O_2_) and the third was kept anaerobic (no injection). Ten minutes after this treatment, all vials were purged once again to remove the air from the solution. At this point, a buffer containing reduced MV was added in order to reduce the viable HydA or Fd-HydA. Thereafter, H_2_ measurements were taken from the headspace and analyzed by gas chromatography (the whole experiment was repeated 3 times). The H_2_ production rate of the sample that was kept anaerobic throughout the whole experiment was referred to as the initial activity of the enzyme. The results indicate that upon introduction of 1.7 μmol O_2_ to the reaction, Fd-HydA maintains 25 % of its initial activity, whereas HydA maintains only 7.5 % (*p* < 0.01, *t* test) (Fig. 2). Upon higher oxygen levels of 3.4 μmol O_2_, the fusion enzyme still maintains 15 % of its initial activity as opposed to the native enzyme that shows only a negligible activity of less than 2 % of its initial activity (*p* < 0.01, *t* test) (Fig. 2).

**Fig. 3:**
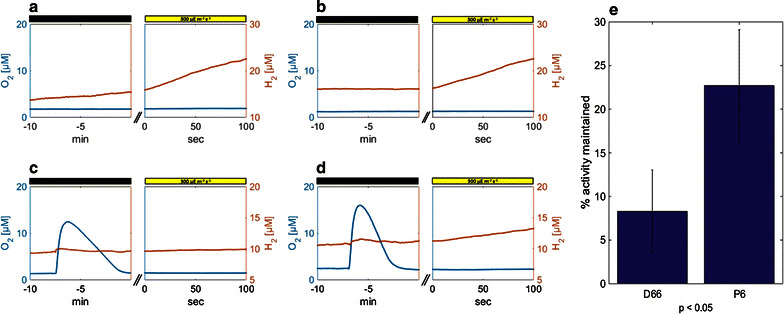
Elevated oxygen tolerance of the fusion enzyme in vivo. **a** O_2_ (*blue*) and H_2_ (*orange*) continuous measurements for wt strain D66, as measured by MIMS. The *left window* depicts the last 10 min of a dark anaerobic incubation (out of 60 min overall), whereas the *right window* refers to the first 100 s of illumination immediately following the incubation. **b** The same analysis as in (**a**) for the Fd-HydA expressing clone P6. **c** The same analysis as in (**a**), with a 500-µL injection of aerobic TAP-HEPES 7 min prior to illumination. **d** The same analysis as in **c** for the Fd-HydA expressing clone P6. **e** Survival percentage is calculated by dividing the hydrogen production rates obtained after oxygen injection (seen in *panels* **c**, **d**) by the matching H_2_ production rates obtained in anaerobiosis (seen in *panels* **a**, **b**). The whole experiment was repeated 3 times (biological repeats). *Panels* (**a**–**d**) each depict the results of a single experiment, whereas the data shown in **e** and the calculated *p* value (*p* < 0.05) refer to the mean over all 3 repeats

To study whether this O_2_-tolerance trait of the fusion enzyme exists in vivo as well, we closely studied the effect of O_2_ on photosynthetic H_2_ production in our highest Fd-HydA expressing clone, P6, and in its parental strain, the HydA expressing wt D66. Initially, we verified that these two clones share similar overall photosynthetic behavior as well as photoheterotrophic, photoautotrophic and dark heterotrophic growth rates and ${\text{ETR}}_{\text{aerobic/anaerobic}}^{{^{\text{PSII}} }}$ (Additional file 7 panels a–d). After verifying that P6 and D66 have similar overall phenotypic behavior, an in vivo photosynthetic assay was conducted and recorded using membrane inlet mass spectrometer (MIMS) (Fig. 3). This instrument provides a continuous and simultaneous measurement of both H_2_ and O_2_ concentrations within a liquid cell culture. Cell cultures in mid-log phase (3 × 10^6^ cells mL^−1^) were taken from each of the strains, concentrated and re-suspended in 5 mL TAP-HEPES, to reach a final concentration of 15 μg (chl) mL^−1^. The cells were then incubated in the dark within the MIMS measuring vial for 60 min. The vial was filled completely with cell culture to avoid any headspace. During the incubation period, the initial O_2_ concentrations gradually decrease due to cellular respiration until anaerobiosis is achieved (Additional file 7). At the end of this period (Fig. 3a, b; left panels), the cells are illuminated with red LED actinic light of 300 μE m^−2^ s^−1^ (Fig. 3a, b; right panels), H_2_ starts accumulating in the solution and the hydrogen production rate is determined (the rate measured under these conditions is referred to as the initial rate).

In a subsequent measurement, a second sample was drawn from the same culture and incubated in the same manner. This time, after 53 min of dark incubation, 500 μL of aerobic TAP-HEPES media were injected into the anaerobic solution (reaching a final concentration of 18 µM O_2_). During the next 6–7 min of dark incubation (Fig. 3c, d; left panels), O_2_ levels plummet (due to homogenous constant mixing and natural respiration) and anaerobiosis is re-achieved. As in the first measurement, the culture was exposed to illumination of 300 μE m^−2^ s^−1^ after a total of 60 min of incubation and hydrogen production rates were measured (Fig. 3c, d; right panels). In the second measurement, H_2_ accumulation was visibly slower and the percentage of maintained activity was calculated by dividing the recorded rates by the initial hydrogen production rates (this experiment was repeated 3 times for each of the two clones). The results (Fig. 3e) show that P6, expressing Fd-HydA, maintained 23 % of its initial hydrogen production activity, whereas D66 expressing endogenous HydA, maintained only 7 % (*p* < 0.05, *t* test).

## Discussion

In the current work we dealt with two of the main challenges facing the engineering of a hydrogen producing micro-algal strain: (i) inefficient electron transfer to hydrogenase and (ii) oxygen sensitivity. We expressed the Fd-HydA fusion enzyme in *C. reinhardtii* by nuclear transformation. Using two different transformants, P6 (psaD promoter) and 1D4 (Hsp70A-RbcS2 promoter), both expressing the Fd-HydA at different levels, we provide evidence that our synthetic enzyme’s in vivo H_2_ production rate is 4.5-fold greater than that of the native HydA.

It should be noted that these results support our previous published work where we showed in vitro that the Fd-HydA fusion results in an increased photosynthetic hydrogen production rate [9]. Thus, these results further support our hypothesis that “the localization of Fd-HydA at the proximity of PSI results in an enhanced electron transfer”. Interestingly, the PHPRH of neither HydA nor Fd-HydA is dependent on the amount of the total cellular enzyme contents. Rather, the increased PHPRH of Fd-HydA is an intrinsic feature resulting from the fusion’s ability to better acquire electrons at the expense of FNR, as we previously showed in vitro [9]. However, since natural expression levels of HydA in the wt are an order of magnitude greater than the current expression levels of the fusion, better expression of synthetic enzymes must be achieved in order to reach optimal total activity. This could be achieved by utilizing plastid transformation where much higher amounts of transgene can be expressed as was recently shown [23].

In the second part of this study we have seen a partial improvement in oxygen tolerance of the engineered Fd-HydA fusion protein. This tolerance was initially shown in vitro for the purified Fd-HydA (Fig. 2) and was further shown in vivo (Fig. 3). Hence, these results imply that the fusion of the Fd moiety which contains an additional 2FeS cluster, either (i) directly functions as bait for oxygen molecules, i.e., reducing molecular oxygen to superoxide [22] which due to its charge cannot penetrate into the active site of HydA, or (ii) partially blocks the oxygen pathway leading to the active site of HydA. On the top of that, the Fd moiety tethers the Fd-HydA to PSI where a putative local anaerobic environment is formed by a direct oxygen uptake in PSI, also called the “Mehler reaction”, thus resulting in an additional layer of protection from oxygen. All of these proposed mechanisms should be further and thoroughly studied to engineer a better protection from oxygen.

## Conclusions

Our work shows that by using a synthetic biology approach, we could achieve an increased PHPRH for hydrogen production in vivo by employing a synthetic enzyme. The Fd-HydA fusion protein, that was previously shown to efficiently divert electron flow from NADPH to hydrogen production in vitro [9], was shown to be efficient in vivo as well. The Fd moiety of this enzyme not only provides it with better electron acceptance abilities, but also partially protects it from the deleterious effect of oxygen. Hence, a successful high expression of this enzyme together with other reported improvements, e.g., the engineered Fd shown by Winkler et al. [7] in the strain Stm6 [24], could be of utmost benefit for the engineering of futuristic industrial hydrogen producing algal strains.

## Methods

### Algal cultures and growth conditions

*Chlamydomonas reinhardtii* wild-type strain CC-124 (nit-, mt-) was obtained from the Chlamydomonas Genetic Center (http://www.chlamy.org/). The *hydA1*-*1 hydA2*-*1* mutant (*hydA*_*1,2*_) and the parental strain D66 were a kind gift from Matthew Posewitz, School of Mines, CO, USA. The strain *hydA*_*1,2*_ was used for generation of transformants. The algal cultures were grown in Tris-Acetate-Phosphate (TAP) medium at 25 °C under continuous cool daylight and cool white fluorescent lights (90 µE m^−2^ s^−1^) stirring in 100-mL Erlenmeyer’s flask capped with silicone sponge enclosures.

### Chlorophyll determination and cell count

The physiological experiments were performed when the cultures reached the density of 3 × 10^6^ cells mL^−1^ (as determined by Celeromics cell counter) that corresponds to chlorophyll concentrations of ~9 µg chl mL^−1^. Chlorophyll was determined according to Arnon [25] using a Cary 50 Bio (Varian) spectrophotometer.

### *C. reinhardtii* transformant generation

Construction of transformants: A *Chlamydomonas* codon-optimized (GeneArt) sequence, coding for the fusion protein ferredoxin [Gly_X4-_Ser]_X3_ hydrogenase (Fd-HydA), was inserted into three vectors, each with a different promoter. In pChlamy_1 expression vector (GeneArt) the fusion sequence was cloned under the Hsp70A-RbcS2 promoter (Additional file 1a). The fusion protein was preceded by the Fd transit peptide sequence. To elevate fusion protein expression, the first intron of the small subunit of ribulose bisphosphate carboxylase (RbcS2) was inserted into the fusion protein coding sequence. A strep-tag sequence and the 3′ UTR from RbcS2 gene were added before and after the stop codon, respectively. The pChlamy_1 vector harbors the *Streptomyces hygroscopicus* aminoglycoside phosphotransferase gene (Aph7), which confers resistance to hygromycin. In pSL18 expression vector the fusion sequence was cloned under the control of the psaD promoter [17] (Additional file 1b). In puC19 expression vector, a kind gift of Prof. Maria Ghirardi (NREL, Golden) the fusion sequence was cloned under the control of the HydA endogenous promoter (Additional file 1c). All constructs were transformed by electroporation into *hydA*_*1,2*_ following the GeneArt Chlamydomonas Engineering Kit protocol (Life Technologies).

### *Rhodobacter capsulatus* quantitative assay

Algal strains, overlaid with engineered H_2_-sensing *R. capsulatus*, were prepared as described previously [19]. The plates were then scanned using the Fuji FLA-5100 fluorescence imager. A 473 nm laser was used for excitation, whereas 510 and 665 nm filters were used for quantifying GFP luminescence and chlorophyll density, respectively. Fluorescence quantification was carried out by using the imager’s software (FLA-5100 image gauge).

### Determination of hydrogenase activity by gas chromatography

Analytic gas chromatography for quantitation of hydrogen gas within gaseous samples was carried in either HP-5890 or 6890 equipped with a TCD detector and a 5A Mol-Sieve column. Argon was used as a carrier gas, hydrogen typically eluted at 0.7 min.

### Anaerobic induction and photosynthetic hydrogenase activity

Anaerobic induction and photosynthetic hydrogenase activity were carried out as described by Meuser et al. [15], with some modifications. Liquid cell cultures (40 mL) at mid-log phase (3 × 10^6^ cells mL^−1^) were concentrated by centrifugation (2800×*g* for 7 min) and re-suspended in 4 mL of anaerobic induction buffer AIB (50 mM potassium phosphate, pH 7.2; 3 mM MgCl_2_). The concentrated cells were transferred into aluminum foil covered, septum stopper sealed 14 mL glass serum vials (Wheaton) and purged for 30 min with Argon at room temperature. Thereafter, the concentrated cultures were incubated in dark, at room temperature, under 60 rpm agitation for additional 90 min. After anaerobic induction, the cultures were transferred to light (300 µE m^−2^ s^−1^) for 15 min under constant stirring and analyzed for H_2_ content by injecting 500 µL (Hamilton) of the headspace into the GC. The photosynthetic hydrogenase activity was expressed in µmol(H_2_) mg(chl)^−1^ h^−1^.

### Chemical measurement of the intrinsic hydrogenase activity

Anaerobic induction was performed as described above. MV assays for hydrogenase activity were performed as described by Meuser et al. [15]. except that the reactions were incubated at 37 **°**C with 160 RPM agitation for 30 min. H_2_ concentration in 500 µL headspace was determined by GC. The chemical hydrogenase activity was expressed in µmol(H_2_) mg(chl)^−1^ h^−1^. The amount of enzyme (ng and µmol) was calculated based on the in vitro specific activity of HydA and Fd-HydA determined previously [9, 20, 21].

### Immunoblot analysis

Soluble proteins were isolated from 200 mL mid-log phase *C. reinhardtii* (3 × 10^6^ cells/mL). After anaerobic induction (see above) the cells were precipitated (3200×*g*, 5 min) and re-suspended in buffer A (50 mM Tris–HCl pH 8.5, 20 mM Na-dithionite, 60 mM NaCl and 1 mM protease inhibitor cocktail). The cell suspension was lysed in a Minilys tissue lyser (Bertin technologies) at three 5000 rpm cycles of 45 s each in the presence of Sigma glass beads (425–600 µm). The soluble proteins were isolated by a 10-min 14,000×*g* centrifugation and the total soluble protein concentration was determined by commercial Bradford solution (BioRad). The concentration varied between 3 and 6 µg/µl depending on the cell type. After addition of sample buffer (Bolt™ LDS Sample Buffer) containing reducing agent and incubation of samples at 95 °C for 4 min, increasing amounts (1–5 µL) of soluble proteins and known amounts (1–5 ng) of heterologously expressed and purified HydA or Fd-HydA were loaded and fractionated by 4–12 % Bis–Tris Plus PAGE gels (Novex by Life technologies) and analyzed by immunoblotting using iBind™ blotter and its specific blocking reagents (Life technologies). As a primary antibody we used a rabbit polyclonal HydA1/2 antibodies (Agrisera). Membrane images were taken using DNR-MicroChemi station and bands intensities were analyzed using the software Gel-Quant for an exact quantification of each band. The size markers were overlaid using the RGB camera included in the DNR.

### Measurement of the effect of O_2_ on purified hydA and Fd-HydA

HydA and Fd-HydA enzymes were expressed and purified from an *E. coli* rosetta strain as described [20]. After the original purification the Fd-HydA and the HydA batches were diluted to a concentration of 0.4 µM (400 nM). For the experiment, a set of three 14-mL septum-sealed glass Wheaton vials were prepared for each type of enzyme. The relevant enzyme stock (100 µL) was diluted with 900 µL of a dilution buffer (100 mM TRIS–HCl pH = 8) to obtain a final concentration of 40 nM. Then the vials were purged for 1 h with argon and incubated overnight in an atmosphere of 97 % N2 and 3 % H_2_). The vials were then purged with argon for 10 min. The next step included the introduction of air into the sealed vials; for each set of 3 vials containing the same type of enzyme, one vial was left untouched, the second vial was injected with 200 µL of air (1.7 µmol O_2_) and the third vial was injected with 400 µL of air (3.4 µmol O_2_). Injections were done by a Hamilton gas tight syringe. All vials were left to stand on the benchtop for 10 min, then purged again with argon for 10 min, injected with 1 mL of MV activity buffer (100 mM TRIS–HCl pH = 8, 20 mM sodium dithionite, 10 mM MV) and finally incubated at 42 **°**C for 30 min. At the end of the incubation period, 500 µL of gas were drawn from the headspace of each vial and injected into a gas chromatograph. The level of O_2_ tolerance was defined as the total activity in a vial that was introduced to air, divided by the total activity in the vial that remained anaerobic throughout the experiment. These experiments were repeated three times.

### MIMS—mass spectrometric analysis of gas exchange in algal cultures

Membrane inlet mass spectrometer (MIMS) was used in order to record oxygen evolution in vivo [26]. For analyses, 5 ml of concentrated sample at 15 µg chl mL^−1^ was introduced to a quartz cuvette (Starna cells) sealed with a silicon septum. A quadrupole mass spectrometer (QMS 200 M1, Pfeiffer Vacuum) was connected to an inlet probe that pierced through a septum head of a quartz cuvette. The cuvette was then fitted into a metabolic chamber (Optical unit ED-101US/MD, Walz LTD.) which kept the sample thermostated at 24.5 °C during the experiments. LED red light illumination was guided to the cuvette through the light ports of the chamber using the Dual-DB module of the dual PAM-100 (Walz LTD.). Masses of H_2_, N, O_2_ and argon were repeatedly measured with 0.5 s dwelling time per mass. O_2_ trace was normalized to the argon trace in order to compensate for the continuous removal of the measured gas by the vacuum line [27], H_2_ signal was normalized and measured as described [28].

### ETR measurement

Pulse-amplitude modulation of chlorophyll-a fluorescence was recorded with a monitoring system (Dual-PAM-100, Heinz Walz GmbH, Germany).

Electron transport rate out of PSII was calculated as follows:

$${\text{Rate}}\; [ \upmu {\text{mol}}\;{\text{m}}^{ - 2} \;{ \sec }^{ - 1} ]\; = \;{\text{PPFD}}*q_{A} *0.5*\emptyset_{P}$$

PPFD is the photon flux density; *q*_*A*_ is the absorbed light coefficient of each sample; 0.5 is the ratio between PSII:PSI; and $\emptyset_{P} = {{\Delta F}}/{Fm^{\prime}}$ the effective quantum yield of PSII during a light period [29]. In order to calculate the *q*_*A*_, light intensity was measured before (*I*_0_) and after (*I*) the cuvette filled with the algal suspension, during illumination with a light meter (Licor-100, Licor Biosciences, USA). The ln(*I*_0_ − *I*) was plotted against dilution series of the algal suspension and the slope (*α*) and intercept were determined with linear regression by the least sum of squares method [30], *q*_*A*_ then equals 1 − exp(−*α*).

### Additional files

**Additional file 1.** Schematic representation of vectors used for *C. reinhardtii* transformation. Fusion vector under the (a) Hsp70-RbcS2 promoter, (b) psaD promoter and (c) endogenous hydA1 promoter.

**Additional file 2.** *R. capsulatus* screen of *C. reinhardtii hydA*_*1,2*_ double mutant cells transformed with the pUC19 Fd-HydA plasmid (Additional file 1c). Chlorophyll fluorescence of the algae is shown in red whereas bright halation represents GFP produced by *R. capsulatus* upon H_2_ presence. No high expression of Fd-HydA was observed for any of the clones expressing this fusion protein under control of the hydA1 endogenous promoter. 1D4 (yellow circles) and *hydA*_*1,2*_ double mutant (blue circles) clones were used as positive and negative controls, respectively.

**Additional file 3.** Anaerobic induction either in light or in dark. Fd-HydA transformants (P6, 1D4) or the wt strain CC-124 were grown to mid-log phase (3 × 10^6^ cells ml^−1^). Each culture (40 mL) was concentrated and re-suspended in 4 mL AIB (see “Methods” section) in 14 mL septum-sealed glass Wheaton vials. The vials were incubated either in light (100 μE m^−2^ s^−1^) or in dark for 60 min under continuous Argon sparging. At the end of the incubation period all cultures were transferred to light (300 μE m^−2^ s^−1^) and the photosynthetic H_2_ production rate was measured by GC after 15 min of illumination, as described in the “Methods” section.

**Additional file 4.** Photosynthetic H_2_ production rates as a function of increasing anaerobic induction time. Photosynthetic H_2_ production rates were measured for wt strains CC-124 and D66 and for the Fd-HydA expressing clones P6 and 1D4 as described in the methods section (*Anaerobic induction and photosynthetic hydrogenase activity*). Hydrogen production rates for all clones increase gradually during the first 2 h of incubation in dark anaerobiosis. After roughly 2 h all clones reach a plateau where further incubation time does not increase hydrogen production rates. This measurement was repeated 3 times (biological repeats) with the same conclusion each time. Shown here is one of the repeats.

**Additional file 5.** Determination of cellular starch content was performed as described in [31] (Fouchard et al., [31]), using a commercial kit (K-TSHK; Megazyme). Cell cultures were grown to mid-log phase (as described in “Methods” section), then divided into 2 groups; the first group was measured immediately and the second was measured after 2 h of dark anaerobic incubation (as described in “Methods” section). The margin shown is the mean over 3 biological repeats for each clone. Each repeat was simply calculated: Δ = (starch content before induction) − (starch content after induction). The mean recorded values were - 1D4 before induction: 45 mg(starch) mg(chl)^−1^, 1D4 after induction: 37.3 mg(starch) mg(chl)^−1^. P6 before induction: 59 mg(starch) mg(chl)^−1^, P6 after induction: 52.1 mg(starch) mg(chl)^−1^. D66 before induction: 40.9 mg(starch) mg(chl)^−1^, D66 after induction: 35.8 mg(starch) mg(chl)^−1^. CC-124 before induction: 25.8 mg(starch) mg(chl)^−1^, CC-124 after induction: 19.4 mg(starch) mg(chl)^−1^.

**Additional file 6.** Verification of the MV quantification assay by immunoblot quantification. Mid-log phase *C. reinhardtii* cells (200 mL of 3 × 10^6^ cells mL^−1^) were induced anaerobically for 120 min and subsequently analyzed by MV assay and by immunoblotting as described in the methods section. (a**)** An immunoblot of the transformant P6. The lanes marked S1, S2 and S3 contained 16, 32 and 48 µg total soluble protein, respectively. The lanes marked 1, 2, 3, 4 contained 1, 2, 3, 4 ng purified Fd-HydA (panel a) or HydA (panel b) standards co-loaded with 20 µg *hydA*_1,2_ soluble proteins. Numbers with superscript stars (e.g., 1*) represent standards loaded without double mutant *hydA*_1,2_ soluble protein. (b) An immunoblot of D66. The lanes marked S1, S2, S3, S4 contained 14, 17, 10, 15 µg total soluble protein, respectively. (c) Comparison of the enzyme amounts (µmol enzyme/mg chlorophyll) determined by the MV assay (yellow) *versus* the immunoblot (blue).

**Additional file 7.** Comparison of phenotypic features of D66, P6 and *hydA*_1,2_. (a) Photoheterotrophic growth curves in TAP, under 90 μE m^−2^ s^−1^ and constant stirring. (b) Photoautotrophic growth curves in TP (medium lacking organic carbon), under 90 μE m^−2^ s^−1^ and constant stirring. (c) Heterotrophic growth in dark, assayed on TAP plates (1.5 % Difco agar). The picture was taken 4 days after plating. (d) ETR of all clones before (aerobic) and after 2 h of anaerobic induction (see “Methods” section).

**Additional file 8.** MIMS oxygen traces recorded during 1 h of dark anaerobic incubation of D66 and P6 prior to turning the light for the experiments depicted in Fig. 3.

## Abbreviations

HydA: algal [FeFe]-hydrogenase
Fd: ferredoxin
FNR: ferredoxin-NADP^+^-reductase
Fd-HydA: ferredoxin-[FeFe]-hydrogenase fusion protein
PSI: photosystem-I
PHPRH: photosynthetic hydrogen production rate standardized for hydrogenase amount
MV: methyl viologen
MIMS: membrane inlet mass spectrometer
PAM: pulse-amplitude modulated fluorometer
