# Supplementary figures and images for: The dual effect of a ferredoxin-hydrogenase fusion protein in vivo: successful divergence of the photosynthetic electron flux towards hydrogen production and elevated oxygen tolerance
Source: Biotechnol Biofuels. 2016 Aug 30;9:182. doi: 10.1186/s13068-016-0601-3 (PMC5006448; doi:10.1186/s13068-016-0601-3)

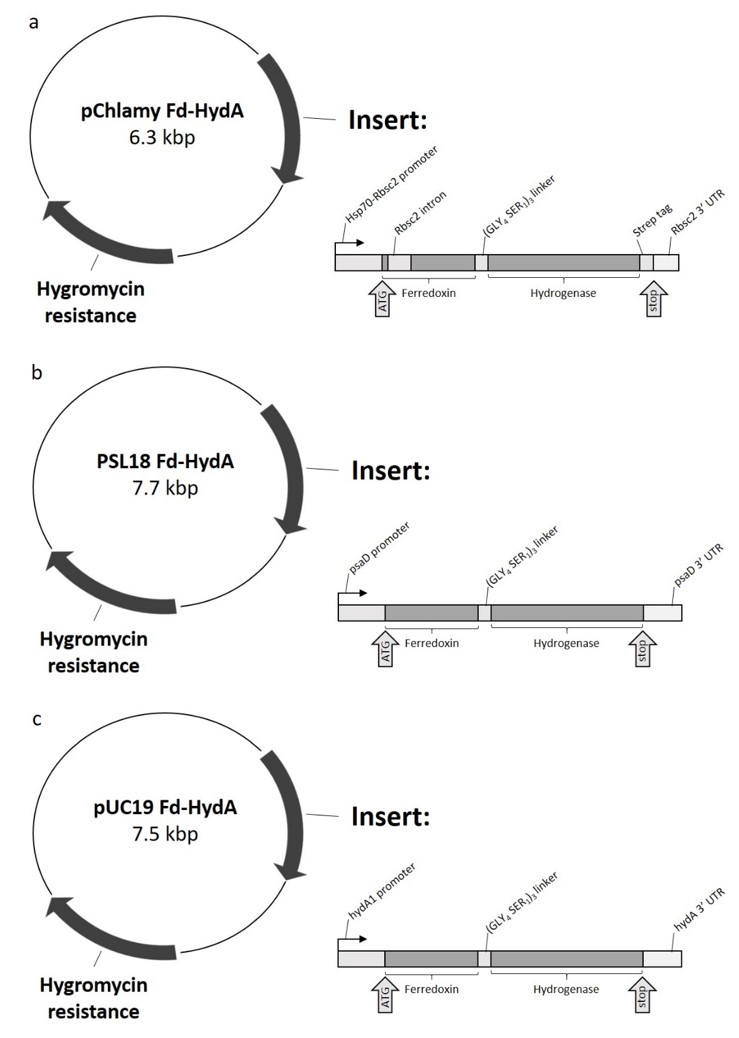

Supplement: Supplementary file 1 — Additional file 1. Schematic representation of vectors used for C. reinhardtii transformation. Fusion vector under the (a) Hsp70-RbcS2 promoter, (b) psaD promoter and (c) endogenous hydA1 promoter. [file 13068_2016_601_MOESM1_ESM.jpg]

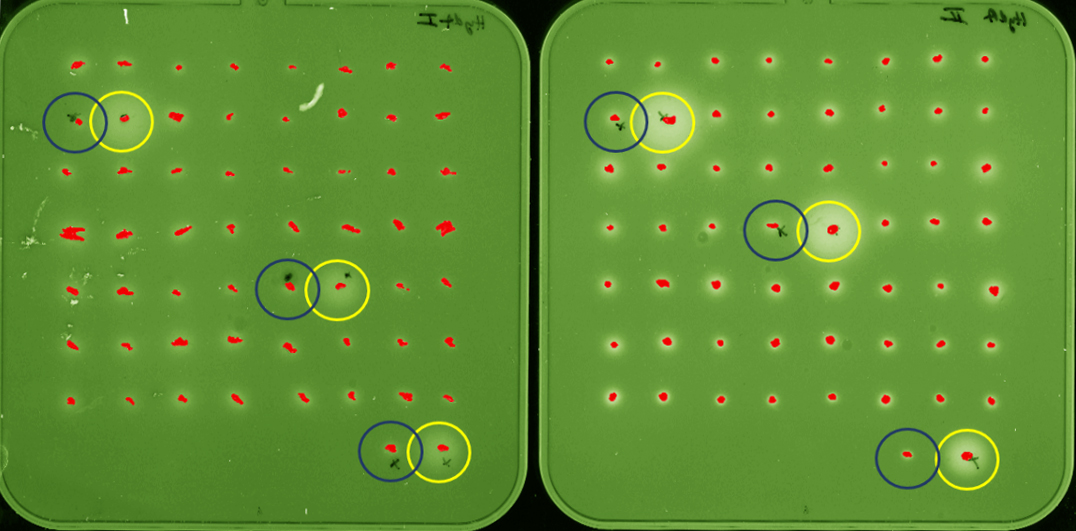

Supplement: Supplementary file 2 — Additional file 2. R. capsulatus screen of C. reinhardtii hydA 1,2 double mutant cells transformed with the pUC19 Fd-HydA plasmid (Additional file 1c). Chlorophyll fluorescence of the algae is shown in red whereas bright halation represents GFP produced by R. capsulatus upon H2 presence. No high expression of Fd-HydA was observed for any of the clones expressing this fusion protein under control of the hydA1 endogenous promoter. 1D4 (yellow circles) and hydA 1,2 double mutant (blue circles) clones were used as positive and negative controls, respectively. [file 13068_2016_601_MOESM2_ESM.jpg]

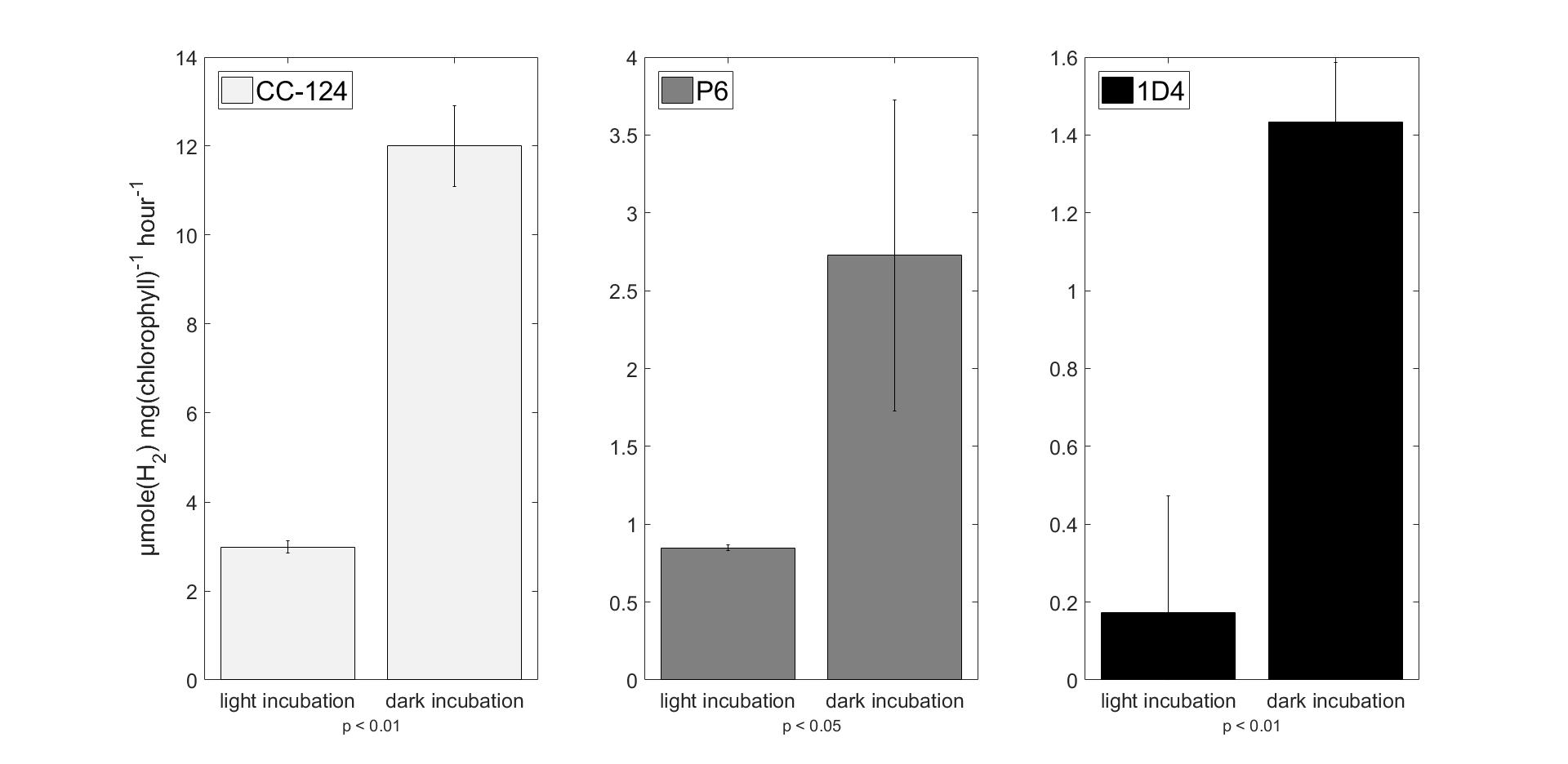

Supplement: Supplementary file 3 — Additional file 3. Anaerobic induction either in light or in dark. Fd-HydA transformants (P6, 1D4) or the wt strain CC-124 were grown to mid-log phase (3 × 10^6^ cells ml−1). Each culture (40 mL) was concentrated and re-suspended in 4 mL AIB (see “Methods” section) in 14 mL septum-sealed glass Wheaton vials. The vials were incubated either in light (100 μE m−2 s−1) or in dark for 60 min under continuous Argon sparging. At the end of the incubation period all cultures were transferred to light (300 μE m−2 s−1) and the photosynthetic H2 production rate was measured by GC after 15 min of illumination, as described in the “Methods” section. [file 13068_2016_601_MOESM3_ESM.jpg]

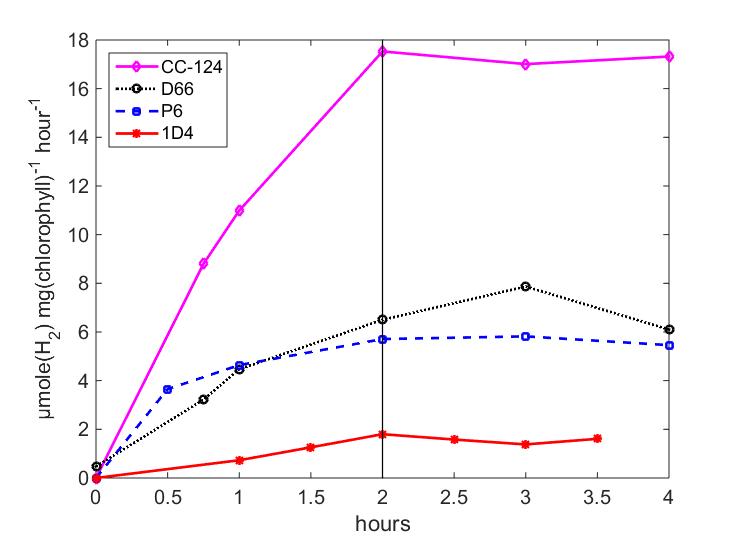

Supplement: Supplementary file 4 — Additional file 4. Photosynthetic H2 production rates as a function of increasing anaerobic induction time. Photosynthetic H2 production rates were measured for wt strains CC-124 and D66 and for the Fd-HydA expressing clones P6 and 1D4 as described in the methods section (Anaerobic induction and photosynthetic hydrogenase activity). Hydrogen production rates for all clones increase gradually during the first 2 h of incubation in dark anaerobiosis. After roughly 2 h all clones reach a plateau where further incubation time does not increase hydrogen production rates. This measurement was repeated 3 times (biological repeats) with the same conclusion each time. Shown here is one of the repeats. [file 13068_2016_601_MOESM4_ESM.jpg]

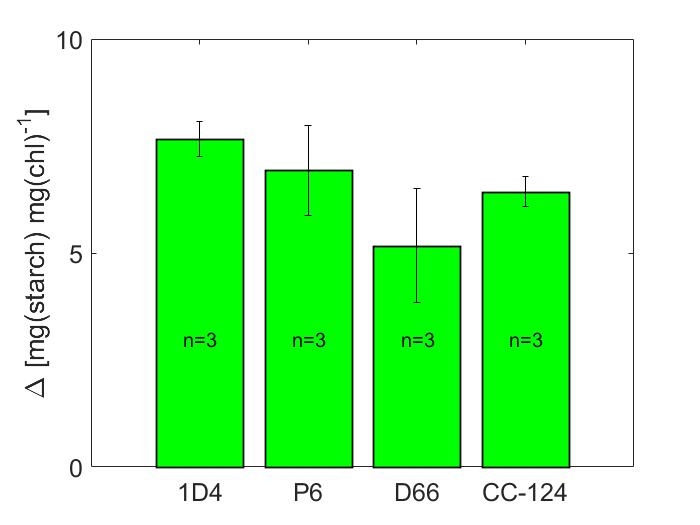

Supplement: Supplementary file 5 — Additional file 5. Determination of cellular starch content was performed as described in [31] (Fouchard et al., [31]), using a commercial kit (K-TSHK; Megazyme). Cell cultures were grown to mid-log phase (as described in “Methods” section), then divided into 2 groups; the first group was measured immediately and the second was measured after 2 h of dark anaerobic incubation (as described in “Methods” section). The margin shown is the mean over 3 biological repeats for each clone. Each repeat was simply calculated: Δ = (starch content before induction) − (starch content after induction). The mean recorded values were - 1D4 before induction: 45 mg(starch) mg(chl)−1, 1D4 after induction: 37.3 mg(starch) mg(chl)−1. P6 before induction: 59 mg(starch) mg(chl)−1, P6 after induction: 52.1 mg(starch) mg(chl)−1. D66 before induction: 40.9 mg(starch) mg(chl)−1, D66 after induction: 35.8 mg(starch) mg(chl)−1. CC-124 before induction: 25.8 mg(starch) mg(chl)−1, CC-124 after induction: 19.4 mg(starch) mg(chl)−1. [file 13068_2016_601_MOESM5_ESM.jpg]

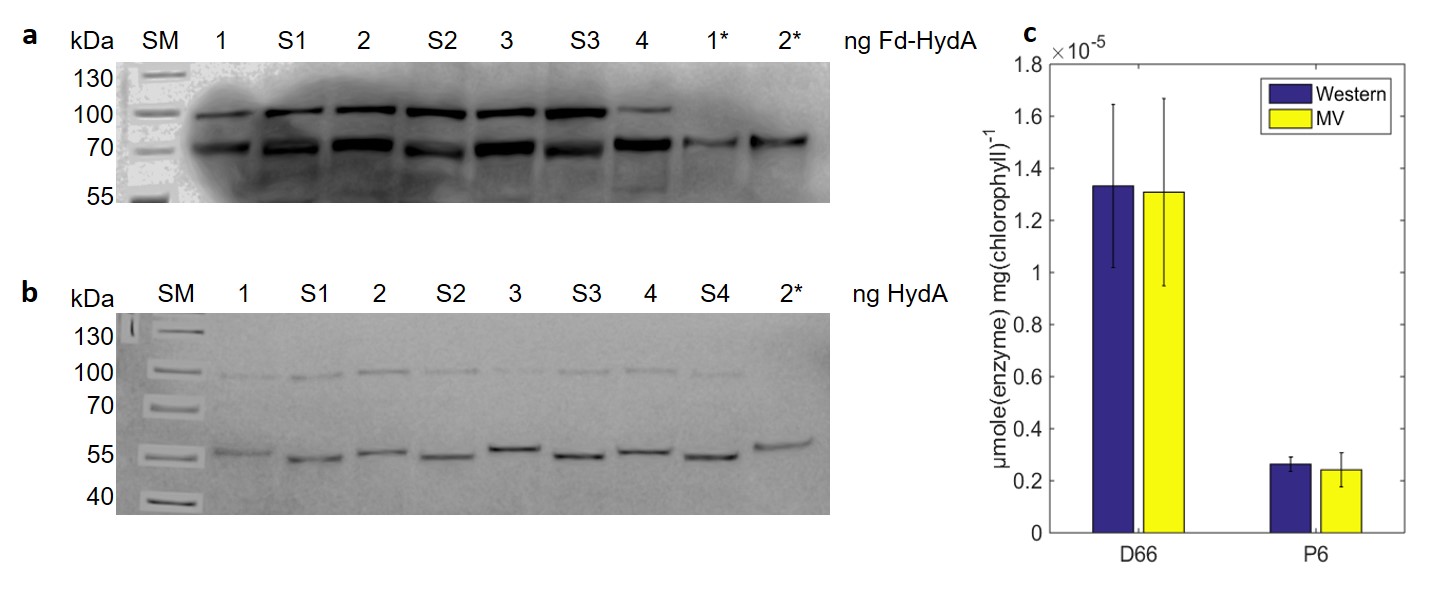

Supplement: Supplementary file 6 — Additional file 6. Verification of the MV quantification assay by immunoblot quantification. Mid-log phase C. reinhardtii cells (200 mL of 3 × 10^6^ cells mL−1) were induced anaerobically for 120 min and subsequently analyzed by MV assay and by immunoblotting as described in the methods section. (a) An immunoblot of the transformant P6. The lanes marked S1, S2 and S3 contained 16, 32 and 48 µg total soluble protein, respectively. The lanes marked 1, 2, 3, 4 contained 1, 2, 3, 4 ng purified Fd-HydA (panel a) or HydA (panel b) standards co-loaded with 20 µg hydA 1,2 soluble proteins. Numbers with superscript stars (e.g., 1*) represent standards loaded without double mutant hydA 1,2 soluble protein. (b) An immunoblot of D66. The lanes marked S1, S2, S3, S4 contained 14, 17, 10, 15 µg total soluble protein, respectively. (c) Comparison of the enzyme amounts (µmol enzyme/mg chlorophyll) determined by the MV assay (yellow) versus the immunoblot (blue). [file 13068_2016_601_MOESM6_ESM.jpg]

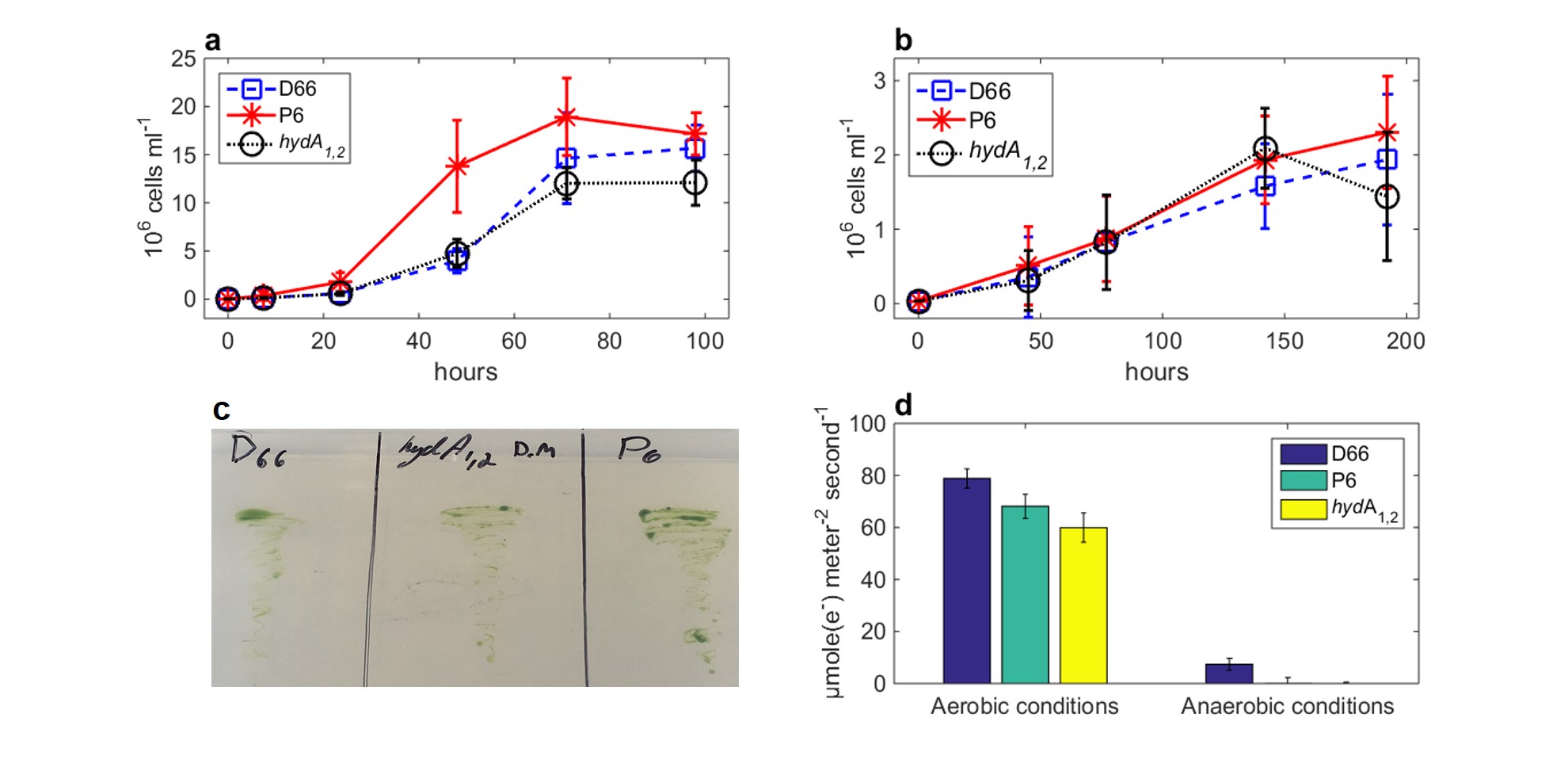

Supplement: Supplementary file 7 — Additional file 7. Comparison of phenotypic features of D66, P6 and hydA 1,2. (a) Photoheterotrophic growth curves in TAP, under 90 μE m−2 s−1 and constant stirring. (b) Photoautotrophic growth curves in TP (medium lacking organic carbon), under 90 μE m−2 s−1 and constant stirring. (c) Heterotrophic growth in dark, assayed on TAP plates (1.5 % Difco agar). The picture was taken 4 days after plating. (d) ETR of all clones before (aerobic) and after 2 h of anaerobic induction (see “Methods” section). [file 13068_2016_601_MOESM7_ESM.jpg]

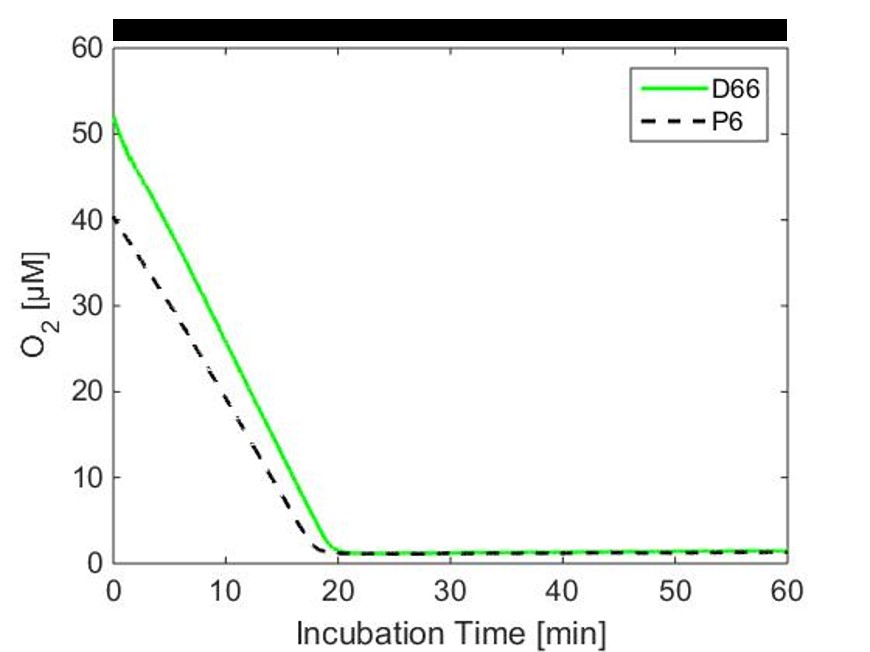

Supplement: Supplementary file 8 — Additional file 8. MIMS oxygen traces recorded during 1 h of dark anaerobic incubation of D66 and P6 prior to turning the light for the experiments depicted in Fig. 3. [file 13068_2016_601_MOESM8_ESM.jpg]
